# Contrasting Vaginal Bacterial Communities Between Estrus and Non-estrus of Giant Pandas (*Ailuropoda melanoleuca*)

**DOI:** 10.3389/fmicb.2021.707548

**Published:** 2021-09-07

**Authors:** Chanjuan Yue, Xue Luo, Xiaoping Ma, Dongsheng Zhang, Xia Yan, Zeshuai Deng, Yunli Li, Yuliang Liu, Junhui An, Xueyang Fan, Lin Li, Xiaoyan Su, Rong Hou, Suizhong Cao, Songrui Liu

**Affiliations:** ^1^Chengdu Research Base of Giant Panda Breeding, Sichuan Key Laboratory of Conservation Biology for Endangered Wildlife, Sichuan Academy of Giant Panda, Chengdu, China; ^2^College of Veterinary Medicine, Sichuan Agricultural University, Chengdu, China

**Keywords:** giant panda, vagina, 16S rRNA gene, microbiome, bacterial community

## Abstract

Bacterial infection and imbalance of bacterial community in the genitourinary system of giant panda could affect the reproductive health. In severe cases, it can also lead to abortion. In this study, 13 of vaginal secretions in the estrue (E) group and seven of vaginal secretions in the non-estrue (NE) group were used to study the composition and diversity of vaginal bacterial communities between estrus and non-estrus by 16S rRNA gene sequencing analysis. The results showed that the vaginal microbiome in giant pandas shared the same top five abundant species between estrus and non-estrus at the phylum level. However, the vaginal microbiome changed significantly during estrus at the genus level. In top 10 genera, the abundance of *Escherichia*, *Streptococcus*, and *Bacteroides* in the E group was significantly higher than that in the NE group (*p*<0.05); *Azomonas*, *Porphyromonas*, *Prevotella*, *Campylobacter*, and *Peptoniphilus* in the NE group was significantly higher than that in the E group (*p*<0.05). The richness and diversity of vaginal microbiome in giant panda on estrus were significantly lower than those on non-estrus (*p*<0.05). It is noteworthy that the abundance of *Streptococcus*, *Escherichia*, and *Bacteroides* of vagina in giant pandas maintained low abundance in the daily. Whereas, they increased significantly during estrus period, which may play an important role in female giant pandas during estrus period. It was hypothesized that hormones may be responsible for the changes in the vaginal microbiome of giant pandas between estrus and no-estrus stages.

## Introduction

The community of microorganisms associated with the host have been shown to affect physiology, immunity and metabolism (Smith and Ravel, 2017). In vagina, microbes exist in co-operative relationship with the host and provide the first line of defense against the migration of opportunistic pathogens. Previous researches have indicated that the imbalance of the vaginal bacteria maybe implicated in genital tract infections, even can result in reproductive failure (Stumpf et al., 2013; Payne and Bayatibojakhi, 2014). Thus, it is important to study the composition of bacterial microbiome and compare the diversity of bacterial communities of the giant panda’s vagina on estrus and non-estrus, which may aid in the prevention and treatment of genital tract infections and negative pregnancy outcomes.

The giant panda (*Ailuropoda melanoleuca*), as a flagship species for wildlife conservation, is a national treasure in China and also well-known in the world (Peng et al., 2007). As an umbrella species, it protects other endangered wildlife in the same nature reserve. Since the giant panda population remains threatened by environmental and anthropogenic pressure (Wei et al., 2017), maintaining stable development of the population has been the prior conservation work. Moreover, the genital health is crucial to the giant panda breeding, which is the key to keep population growth, thus the research of genital tract cannot be ignored.

So far, the major vaginal microbiome studies carried out conventional culture-dependent method, biochemical identification method and high-throughput sequencing method. However, the first two methods could not illustrate the vaginal microbiome of giant panda, since they left many uncultured and unidentified bacteria. Considering the cost, accuracy, efficiency and other factors, 16S rRNA gene sequencing analysis was applied to investigate the vaginal microbiome of giant panda. Recently, the study of composition and diversity of vaginal bacterial communities in women and animals used high-throughput sequencing of 16S rRNA gene sequencing method (Clemmons et al., 2017; Smith and Ravel, 2017).

The composition of vaginal microbiome varies in human and other animals. In human, *Lactobacillus* spp., *Atopobium vaginae*, and *Streptococcus* spp. were considered to be major microbiome in the vagina (Yamamoto et al., 2009). *Lactobacillus* spp. have been consistently linked with good vaginal health (Verhelst et al., 2004). The composition of vaginal bacterial communities is different among cows, which is affected by various factors such as species, fitness, geographical distance and differences in animal handling (Otero et al., 2000; Giannattasio-Ferraz et al., 2019). There are few records on the bacterial communities of giant panda’s vagina, mainly focusing on the differences of composition and diversity between vagina and uterine (Yang et al., 2017), as well as the differences in region and age (Zhang et al., 2020). In this study, our aim is to examine the composition and diversity of bacterial communities between estrus and non-estrus in giant pandas’ vagina by using high-throughput sequencing. It helps to better understand the composition and dynamics of the vaginal microbiome, and the factors associated with the effect of estrus on bacterial communities of giant panda.

## Materials and Methods

### Sample Collection

In this study, the samples of giant panda were collected from Chengdu Research Base of Giant Panda Breeding from September 2019 to April 2020. The giant pandas are all 6 to 26-year-old female adult individuals. The animal handling procedures including anesthesia and sampling were approved by Institutional Animal Care and Use Committee (IACUC) of the Chengdu Research Base of Giant Panda Breeding (No. 2019006) for giant panda. The disposable swabs were extended into the vagina of female giant panda to take vaginal secretion samples. A total of 13 samples of vaginal secretions of giant pandas were collected with artificial insemination operation on estrus and, respectively, named E1–E13 belong to the estrus (E) group. The other seven samples were collected on non-estrus when the giant pandas were carried out other examination and, respectively, named NE1–NE7 belong to the non-estrus (NE) group. The summary of the giant panda was shown in Table 1.

**Table 1:** Summary of studied giant panda females at Chengdu Research Base of Giant Panda Breeding, Chengdu, Sichuan, People’s Republic of China.

Samples	Age (yr)	The individual is the same between two groups	No. of cubs in 2020	Date of birth
E1	6y	None	None	
E2	6y	None	None	
E3	9y	None	1	August 16, 2020
E4	10y	NE1	None	
E5	10y	None	None	
E6	11y	None	None	
E7	12y	None	None	
E8	12y	NE2	None	
E9	12y	None	1	July 6, 2020
E10	13y	NE3	None	
E11	15y	None	None	
E12	17y	None	None	
E13	19y	NE6	None	
NE1	10y	E4	None	
NE2	12y	E8	None	
NE3	13y	E10	None	
NE4	16y	None	None	
NE5	19y	None	2	July 4, 2020
NE6	19y	E13	None	
NE7	26y	None	None	

### Methods

#### DNA Extraction of Bacteria

A total of 20 samples of vaginal secretions of giant pandas were collected in this experiment. Thirteen samples of them on estrus and seven samples of them in non-estrus were sent to BGI Co., Ltd., China (Shenzhen, China) for DNA extraction and 16S rRNA gene sequencing analysis. Bacterial DNA extraction was carried out according to the requirements of BGI Co., Ltd., China (Shenzhen, China).

#### PCR Amplification of 16S rRNA V4 Region

The 16S rRNA gene sequencing targeting V4 hypervariable region was amplified from samples of vaginal secretion. In brief, the primers 515F (5'-GTGCCAGCMGCCGCGGTAA-3') and 806R (5'-GGACTACHVGGGTWTCTAAT-3') were designed to amplify the V4 of 16S rRNA gene sequence from the single vaginal microbiome sample. The targeted gene was amplified from the template in PCR mixtures containing PCR master mix, mixture of V4 PCR primers and genomic DNA which was normalized to 30ng per PCR reaction. The melting temperature was 56°C and PCR cycle was 30. Then the PCR amplification products were purified by Agencourt AMPure XP magnetic beads, and dissolved in elution buffer, then completed library construction on HiSeq 2500 platform and the sequencing type is EP250. Agilent 2100 Bioanalyzer was used to detect the fragment range and concentration of the library.

#### Bioinformatics Analysis

To get the clean data, the raw data was filtered to eliminate adapter contamination and low-quality readings with following methods: FLASH (fast length adjustment of short reads, v1.2.11) was used to merge the coincident clean paired-end reads into the tag (Magoc and Salzberg, 2011). Then, the DADA2 (Divisive Amplicon Denoising Algorithm) method in Qiime2 v1.8.0 was used to de-noising and obtain the Amplicon Sequence Variant (ASVs) where ASV is 100% similar sequence, and the Ribosomal Database Project (RDP) Database v.2.2 was used to classify and sort the representative sequences of OTUs (Caporaso et al., 2010). The 16S rRNA sequences data have been submitted to the GenBank databases under accession numbers: SAMN19090826-19090845.

#### Statistical Analyses

Finally, the Venn diagram and bar graph of each group was displayed by R software (v3.1.1). The Alpha diversity index was calculated by Mother (v.1.31.2), and the rarefaction cure was drawn by R software (v3.1.1) to judge the reasonable degree of sequencing data, and the Wilcoxn rank sum test were performed on Alpha diversity index between groups to obtain the difference of species diversity between two groups. UPGMA cluster analysis with weighted Unifrac distance matrix was performed by Phytools and R software (V3.5.1). The cluster tree was used to demonstrate the results. Using iterative algorithm in QIIME (v1.80), in the case of weighted abundance species classification information, sampling analysis is carried out using 75% of the sequence number of all samples with the least sequence number. After 100 iterations, the final statistical analysis result table and Principal Co-ordinates Analysis (PCoA) display diagram are obtained, which are used to study the similarity or dissimilarity of sample community composition between samples. R (v3.4.1) was used to calculate the significance of the difference test between the two groups for the bacteria in the top 10 abundances. LEfSe software^1^ was used to make LEfSe cluster diagram and LEfSe linear discriminant analysis (LDA) diagram. Wilcoxn test was used to look for differences between groups by R (v3.4.1). The function was shown graphically.

## Results

### Sequencing Data

**Figure 1:**
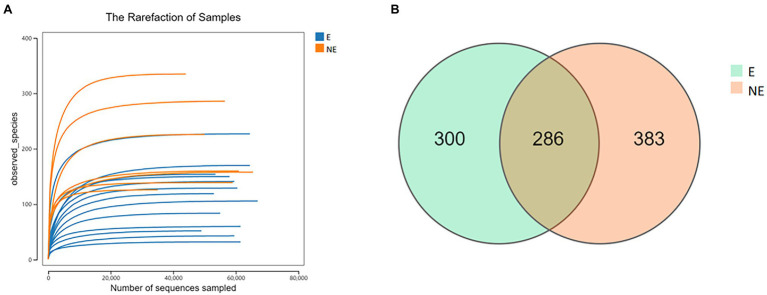
**(A)** Rarefaction curve. Curves of different colors show the observed species (*Y*-axis) of each sample at different number of sequences sampled (*X*-axis). When the curve tended to be flat, the amount of the sequenced data was large enough to reflect the majority of the microbial data in the samples. **(B)** OTU distribution in the two groups. The green and orange circles represented samples of vaginal secretions in the E and the NE group, respectively. The overlap denoted the OTUs shared by two groups.

After the raw data was filtered and spliced, 1,143,725 high-quality tags were produced in the E and NE groups. The sequencing quality was calculated by rarefaction curve. All of the curves were flat and the number of operational taxonomic units (OTUs) was close to saturation (Figure 1A), indicating that the sequencing depth was sufficient to cover all species in the microbiome community. The total numbers of OTUs obtained was 969, among which 286 OTUs were shared by both groups, 300 and 383 OTUs were uniquely identified in the E and the NE groups, respectively (Figure 1B).

### OTU Abundance Analysis

**Figure 2:**
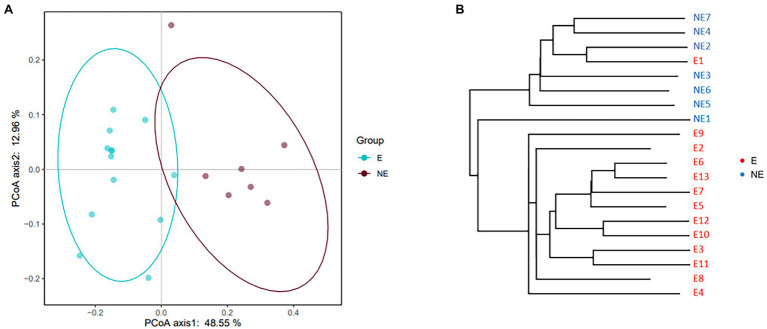
**(A)** PCoA based on the distance matrix (distance other than Euclidean distance) to find principal coordinates. (Description) *X*-axis, PCoA axis1 and Y axis, PCoA axis2. The scale of the *X*-axis and the *Y*-axis are the projection coordinates of the sample points in the two-dimensional plane, respectively. A dot represents each sample, and different colors represent different groups. **(B)** Samples Clustering result (Description, weighted_unifrac). The same color represents the samples in the same group. Short distance between samples represents high similarity.

The result of PCoA showed the clustering (*p*<0.05) was identified for the vaginal secretions of giant panda on estrus and non-estrus (Figure 2A). UPGMA cluster number results also showed that most of the samples in the same group have similar branches (Figure 2B). Futhermore, the results showed an obvious clustering pattern of the E group bacterial community in giant pandas’ vagina, the same as the NE group, indicative of distinct differences between the vaginal microbiome on estrus and that non-estrus of giant pandas.

### Community-Composition Analysis

**Figure 3:**
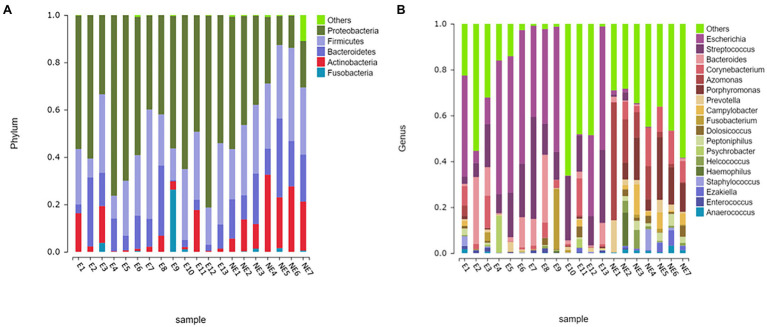
Microbial community barplot. **(A)** Microbial community barplot at the phylum level. Bar charts showing the relative abundance of all genera detected in the vaginal secretions collected from the giant panda in the E (E1–E13) and the NE group (NE1–NE7). The identities of the microbiome were shown with color blocks on the right. **(B)** Microbial community barplot at the genus level. Bar charts showing the relative abundance of all genera detected in the vaginal secretions collected from the giant panda in the E (E1–E13) and the NE (NE1–NE7) group. The identities of the microbiome were shown with color blocks on the right.

**Figure 4:**
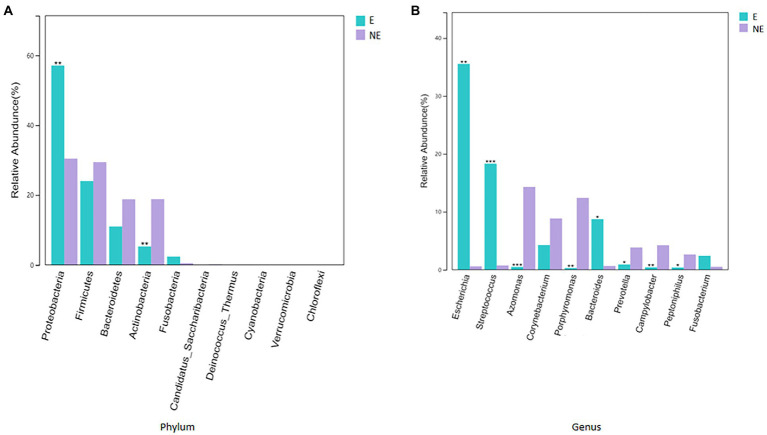
**(A)** Comparison of bacterial differences among different groups of samples at the phylum level in top 10. **(B)** Comparative analysis of different species of bacteria among groups at genus level in top 10 (*p*<0.001, marked with “***”; 0.001<=*p*<=0.01, marked with “**”; 0.01<*p*<=0.05, marked with “*”; if *p*>0.05, not marked).

The taxonomic classification of the sequences from the study samples resulted in 15 different phyla. The sequences that cannot be assigned were categorized as “Unclassified.” When comparing at the phylum level, the NE and E groups shared the same top five bacterial species with little difference in overall abundance. The most dominant phylum of samples in the NE group was *Proteobacteria* (31.6%), followed by *Firmicutes* (29.0%), *Bacteroidetes* (18.8%), *Actinobacteria* (18.2%), and *Fusobacteria* (0.6%; Figure 3A). *Proteobacteria* (57.3%) was also the most abundant phylum in the E samples, followed by *Firmicutes* (24.0%), *Bacteroidetes* (11.1%) and *Actinobacteria* (5.4%) *Fusobacteria* (2.1%; Figure 3A). The estimated cumulative abundance of these five dominant phyla was above 98% of the identified OTUs. The vaginal bacterial community of giant panda in the NE and E groups were similar in composition. However, there was the discrepancy in their relative abundance at phyla level. Further analysis of the relative abundance showed that the abundance of *Proteobacteria* in the E group was significantly higher than that in the NE group (*p*<0.01); whereas, the abundance of *Actinobacteria* in the NE group was significantly higher than that in the E group (*p*<0.01; Figure 4A).

At the genus level, a total of 244 bacterial genera were identified in our study, 171 in the E group and 212 in the NE group. Giant pandas in the the NE group had the highest *Azomonas* (14.8%) content, followed by *Porphyromonas* (12.6%), *Corynebacterium* (8.5%) and *Campylobacter* (4.7%). While giant pandas in the E group displayed the highest amount of *Escherichia*, accounting for 34.5% of the total microbial abundance, the second most abundant was *Streptococcus* (18.2%), followed by *Bacteroides* (8.8%) and *Corynebacterium* (4.3%; Figure 3B). However, the vaginal bacterial communities in the E group of giant pandas were notably different compared to the NE groups and had fewer the number of species. Our results showed that the female giant panda on estrus can lead to changes in the vaginal bacterial community at the genus level.

In accordance with analysis of the relative abundance at genus level, the top 10 species were selected to evaluate the significance of the difference test between the two groups. The results showed that there were differences in *Escherichia*, *Streptococcus*, *Azomonas*, *Porphyromonas*, *Bacteroides*, *Prevotella*, *Campylobacter*, and *Peptoniphilus* between these two groups. The abundance of *Escherichia*, *Streptococcus*, and *Bacteroides* in the E group was significantly higher than that in the NE group (*p*<0.05); *Azomonas*, *Porphyromonas*, *Prevotella*, *Campylobacter*, and *Peptoniphilus* in the NE group was significantly higher than that in the E group (*p*<0.05; Figure 4B).

### LEfSe Analysis

**Figure 5:**
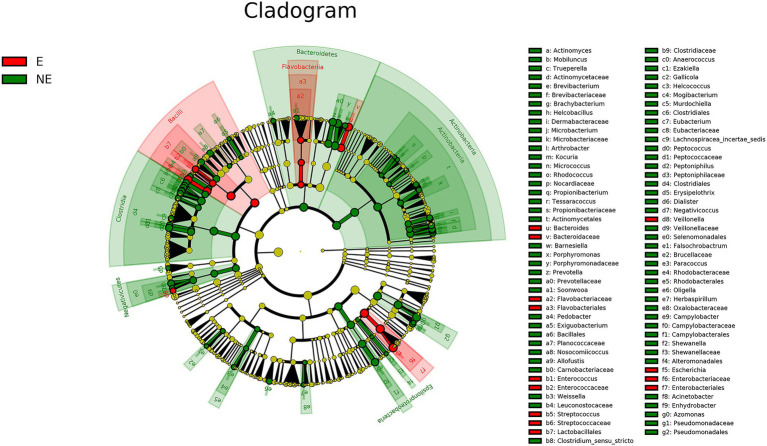
Linear discriminant effect size cluster tree for 16S rRNA gene sequencing analysis. Different colors indicate different groups. Colored notes represent a group, color shading over the notes indicate the significant microbe biomarker in the group causing a significant difference in abundance, and the biomark name is listed in the upper right corner. The yellow notes represent the biomarkers that do not show significant differences in abundance in the groups.

**Figure 6:**
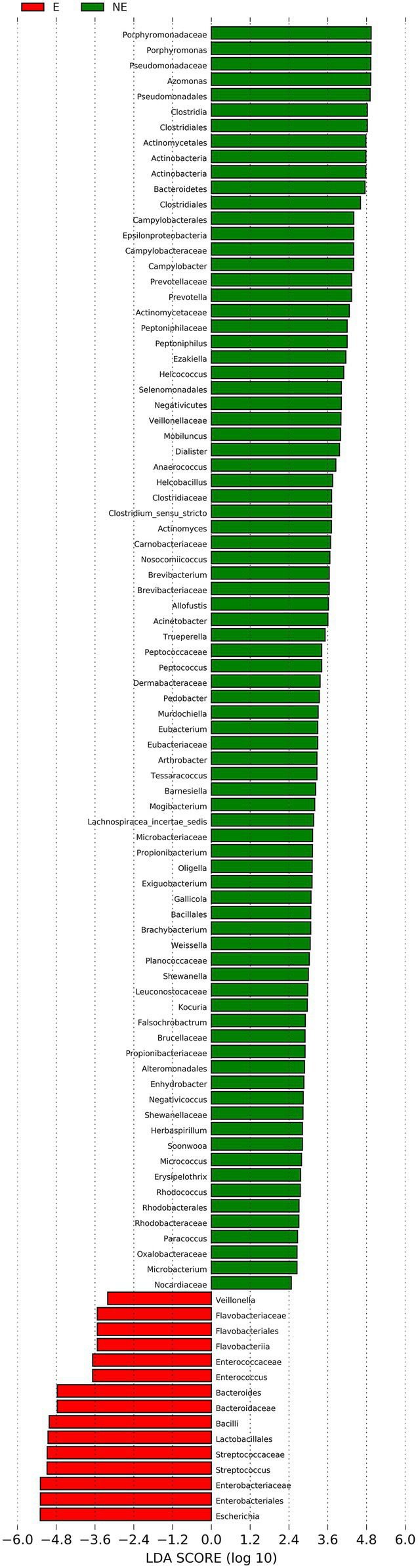
Linear discriminant analysis (LDA) demonstrated distinct bacterial genera enriched in the E group and the NE group. The graph shows the LDA scores obtained from linear regression analysis of the significant microorganism groups in the two groups. When the default LDA value is more than 2 and the *p* value is less than 0.05, the result corresponds to a differential species.

The cladogram of the LEfSe analysis was showed in Figure 5. The green and red parts represented the groups of the NE and E, respectively. The red and green nodes in the cladogram denoted the bacteria playing a critical role in the NE and E group, respectively. The yellow nodes corresponded to the bacteria that did not have an important role in each group. The LDA score was showed in Figure 6, which obtained through LDA. The results showed that the microorganisms were significant difference in the vaginal secretions from giant panda on non-estrus (in the NE group) belonged to *Porphyromonadaceae*, *Porphyromonas*, *Pseudomonadaceae*, *Azomonas*, and *Pseudomonadales*. Morever, the results showed that those were significant difference in the vaginal secretions from giant panda on estrus (in the E group) belonged to *Escherichia*, *Enterobacteriales*, *Enterobacteriaceae Streptococcus*, and *Streptococcaceae*.

### Microbial Diversity Analysis

**Figure 7:**
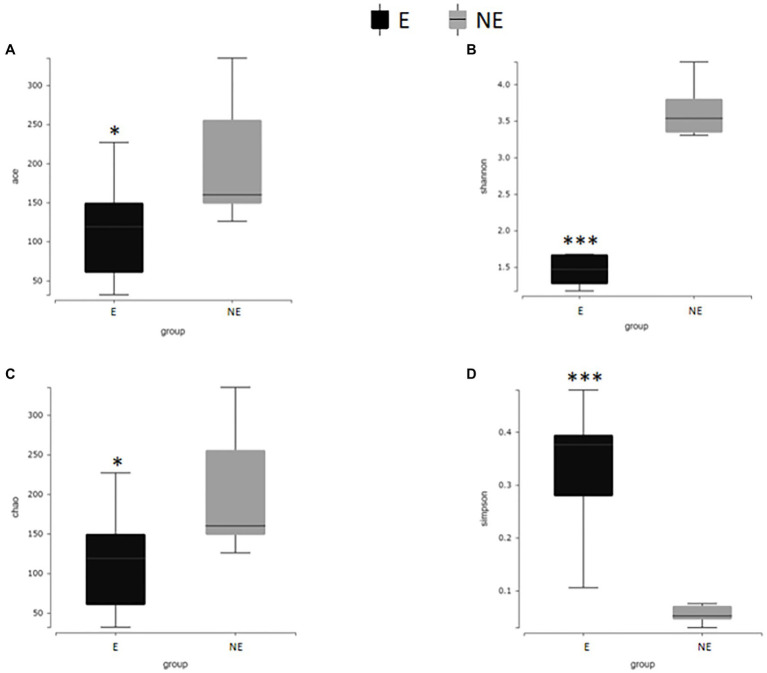
Alpha diversity difference map of vaginal secretions in two groups. **(A)** The comparison result of ACE index; **(B)** the comparison result of Shannon index; **(C)** the comparison result of Chao1 index; **(D)** the comparison result of Simpson index (*p*<0.001, marked with “***”; 0.001<=*p*<=0.01, marked with “**”; 0.01<*p*<=0.05, marked with “*”; if *p*>0.05, not marked).

In Figure 7, the Shannon index, Simpson index, Chao1 index and ACE index of each group were calculated to analyze α-diversity. The calculated ACE index (Figure 7A) and Chao1 index (Figure 7C) were positively associated with the richness of the bacterial species in each group. Our results found that the ACE and Chao1 index in the NE group were significantly higher than that in the E group (*P*_ACE_<0.05; *P*_Chao1_<0.05). These results indicate that estrus reduced the overall number of bacterial species and thus the richness of the vaginal bacterial community in giant pandas.

The calculated Shannon index, (Figure 7B) was positive associated with the diversity of the microbiome, while Simpson index (Figure 7D) was negative associated with the diversity of the microbiome in each group. Our results found that the Shannon index in the NE group were significantly higher than that in the E group (*P*_Shannon_<0.001). The Simpson index in the NE group were significantly lower than that in the E group (*P*_Simpson_<0.001) which indicate that estrus decreases the diversity of the vaginal microbial community in giant pandas.

### Predictive Function Gene Analysis

**Figure 8:**
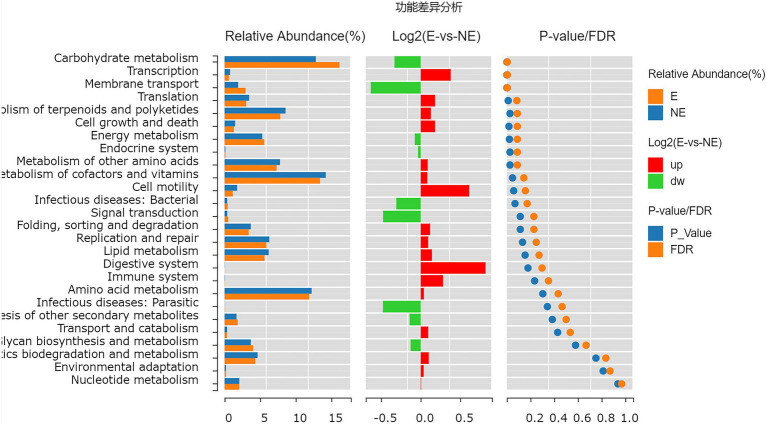
Wilcox Test result map of pathway difference (KEGG level 2). The relative abundance bar chart of each group is shown on the left; the log2 value of the relative abundance mean ratio of the same pathway in the two groups is shown in the middle; and the value of *p* and FDR values obtained by wilcox test are shown on the right. If the value of *p* and FDR values are less than 0.05, the pathway is significantly different between the two groups.

**Figure 9:**
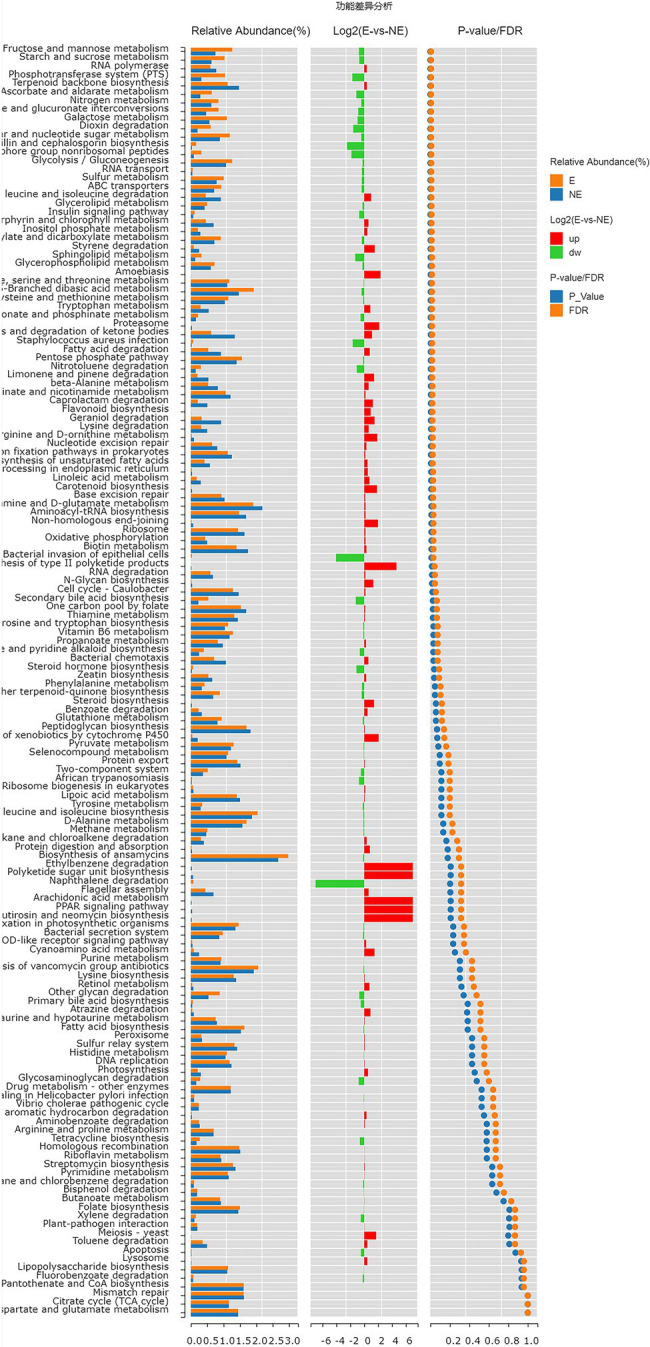
Wilcox Test result map of pathway difference (KEGG level 3). The relative abundance bar chart of each group is shown on the left; the log2 value of the relative abundance mean ratio of the same pathway in the two groups is shown in the middle; and the value of *p* and FDR values obtained by wilcox test are shown on the right. If the value of *p* and FDR values are less than 0.05, the pathway is significantly different between the two groups.

The functional genes of the samples were analyzed by Kyoto Encyclopedia of Genes and Genomes (KEGG) database. The levels of carbohydrate metabolism and membrane transport in the E group were obviously higher than those in the NE group (*p*<0.05; *FDR*<0.05). The level of transcription in the E group was lower than that in the NE group (*p*<0.05; *FDR*<0.05; Figure 8), and the levels of galactose metabolism, phosphotransferase system (PTS), pentose and glucuronate interconversions and so on in the E group were higher than those in the NE group (*p*<0.05; *FDR*<0.05; Figure 9).

## Discussion

There are some reports on the vaginal microbiome composition of giant pandas. However, there is no report to compare the difference of vaginal microbiome of giant panda between estrus and non-estrus according to our knowledge. In this study, we explored the differences about the composition and diversity of giant pandas’ vaginal microbiome between estrus and non-estrus (*p*<0.05). Our results of bacterial composition showed that the vaginal microbiome shared the same top five abundant species between estrus and non-estrus at the phylum level. Whereas, at the genus level, the top 10 abundant bacterial species and their relative abundance were significant difference between estrus and non-estrus. At genus level, microbial diversity and richness of vaginal microbiome of giant panda were decrease during estrus. However the abundance of *Escherichia*, *Streptococcus*, and *Bacteroides* increased and became the main component of the vaginal microbiome as the results showed in Figure 4. The result of LEfSe analysis was consistent with it. It’s hypothesized that the change of microbiome structure makes the vaginal bacterial communities of giant panda more adaptable to the physical changes of giant panda during estrus (Figure 5).

*Streptococcus* was one of main bacteria in the vagina of giant panda during estrus which were significant increased compared with giant panda on non-estrus. Yang et al. (2017) and Zhang et al. (2020) have also report that *Streptococcus* was an important genus of bacteria living in the vagina and uterus of giant pandas on estrus. However, this is the first report describing the change of *Streptococcus* in vagina of giant panda between estrus and non-estrus and illustrated it may play an important role on the estrus of giant pandas. Therefore, it was hypothesized that *Streptococcus* maintained low daily abundance when the giant panda entered estrus. It was significantly increased and performed a lactic acid-producing function, which is similar to the effect of *Lactobacillus* on human. Interesting that *Lactobacillus* was the most common bacteria in human, but its relative abundance was very low in the vaginal microbiome of giant pandas no matter on estrus or on non-estrus which was similar with the result of Yang et al. (2017), moreover *Streptococcus* and *Lactobacillus* belongs to the same orders, *Lactobacillales*.

There are also some reports to support this conclusion. *Streptococcus* probably can decompose glycogen and utilize its degradation products to produce lactic acid and other bacteriostatic substances, which maintains the health of the host by reducing environmental PH (Thomas et al., 1979; Abbott et al., 2010) and other pathways (Gezginc et al., 2015; Wang et al., 2017). In particular, our results of functional prediction showed that the carbohydrate metabolism, galactose metabolism and other level of microorganisms in vaginal secretions on estrus were higher than that on non-estrus, which may associate with the change of *Streptococcus*.

The results of *Escherichia* and *Bacteroides* also interesting. *Escherichia* was the highest amount in the vagina of giant panda during estrus and significantly increased compared with giant panda on non-estrus. It should be one of the important probiotics in the vagina of giant pandas. Other studies have shown that some bacteria belong to *Escherichia* have probiotic effects. It can inhibit the growth of pathogenic microorganisms by metabolizing glucose into acidic products, and competing with other pathogenic bacteria for colonization and other ways (Hejnova et al., 2005; Nag et al., 2018). In the study of Yang et al. (2017), *Escherichia* are also dominant bacteria in the vagina of giant pandas. *Bacteroides* was the third of main bacteria in the vagina of giant panda during estrus. It also significantly increased compared with giant panda on non-estrus. The bacteria belong to *Bacteroides* also have the potential to be probiotics, which can maintain the health of the host by regulating host immunity, competitively inhibiting pathogens and secreting antibacterial substances (Sun et al., 2019).

Hormones may be responsible for the changes in the vaginal microbiome of giant pandas between estrus and no-estrus. Studies have shown that in female human beings, the secretion of estrogen will promote the proliferation of *Lactobacillus* (Pekmezovic et al., 2019). Nugeyre et al. (2019) reported that progesterone can affect the abundance of some microorganisms in the vagina of female cynomolgus macaques. Messman (Messman et al., 2020) reported that estradiol had a certain effect on the bacterial composition of Brangus heifers vagina. However, the corresponding relationship between hormones and vaginal microbiome in giant pandas still needs to be revealed by further study.

## Conclusion

The vaginal microbiome in giant pandas shared the same top five bacterial species between estrus and non-estrus at the phylum level. However, the vaginal microbiome changed significantly during estrus at the genus level. The richness, diversity and the bacterial species at genus level of vaginal microbiome in giant panda on estrus were significantly lower than those on non-estrus. It is noteworthy that the abundance of *Streptococcus*, *Escherichia*, and *Bacteroides* of vagina in giant pandas increased significantly during estrus, which may play an important role in female giant pandas during estrus.

## Data Availability Statement

The datasets presented in this study can be found in online repositories. The names of the repository/repositories and accession number(s) can be found at: https://www.ncbi.nlm.nih.gov/, SAMN19090826-19090845.

## Ethics Statement

The animal study was reviewed and approved by Institutional Animal Care and Use Committee (IACUC) of the Chengdu Research Base of Giant Panda Breeding (No. 2019006).

## Author Contributions

CY, SL, and XM contributed to conception and design of the study. SL and SC plays a guiding role in carrying out the experiment. CY, XL, and XS organized the database. XY, YUL, JA, YLi, and LL collected samples. LX, DS, and ZS performed the statistical analysis. XL and XF wrote the first draft of the manuscript. CY, XL, and RH wrote sections of the manuscript. All authors contributed to manuscript revision, read, and approved the submitted version.

## Funding

This research was supported by Chengdu Research Base of Giant Panda Breeding (project number: 2020CPB-B04), National Forestry and Grassland Administration and Sichuan Provincial Finance Department (project title: study on main epidemiological investigation and prevention of giant panda), and the Double Subject Construction Plan of Sichuan Agricultural University (project number: 03571537).

## Conflict of Interest

The authors declare that the research was conducted in the absence of any commercial or financial relationships that could be construed as a potential conflict of interest.

## Publisher’s Note

All claims expressed in this article are solely those of the authors and do not necessarily represent those of their affiliated organizations, or those of the publisher, the editors and the reviewers. Any product that may be evaluated in this article, or claim that may be made by its manufacturer, is not guaranteed or endorsed by the publisher.

## Footnotes

^1^ https://huttenhower.sph.harvard.edu/galaxy/
